# The role of inhibitory function in associative memory among older adults and its plasticity

**DOI:** 10.1186/s41235-025-00688-5

**Published:** 2025-11-16

**Authors:** Jia-Jie Xu, Jun-Yi Chen, Hong-Zhou Xu, Zhiwei Zheng, Jing Yu

**Affiliations:** 1 Faculty of Psychology, Southwest University, Tiansheng Road, Beibei District, Chongqing, 400715 China; 2 Center on Aging Psychology, State Key Laboratory of Cognitive Science and Mental Health, Institute of Psychology, Chinese Academy of Sciences, No. 16 Lincui Road, Chaoyang District, Beijing, 100101 China; 3 Department of Psychology, University of Chinese Academy of Sciences, Beijing, China; 4 School of Psychology, University of New South Wales, Sydney, NSW Australia; 5 Neuroscience Research Australia, Sydney, NSW Australia

**Keywords:** Associative memory, Gist, Inhibitory function, Acute training, Aging

## Abstract

Associative memory deteriorates with age. One possible reason for this associative memory deficit in older adults is a decline in inhibitory function. However, it remains unclear what role of inhibitory function plays in age-related associative memory deficits, and whether and how acute training of inhibitory function could ameliorate the detrimental effects of inhibitory deficits on associative memory in older adults. In Experiment 1, 80 participants (40 younger and 40 older adults) studied scene-word pairs while attempting to inhibit interfering words during encoding, with two conditions: gist and non-gist interferences. In Experiment 2, 66 older adults were randomly assigned to either acute inhibitory training or a control group, and eye-tracking technology was used to capture the benefits of acute inhibitory training. Results showed that older adults were more disturbed by gist than non-gist interferences because of hyper-binding, and that inhibitory function mediated the relationship between age and associative memory accuracy. Notably, although acute inhibitory training did not significantly improve associative memory accuracy in the training group compared to the control group, structural equation model showed that older adults in the acute training group showed shorter fixation durations and lower frequencies in the interference region of interest, leading to better associative memory. These results indicate that inhibitory function plays a mediating role in age-related associative memory decline, as well as its plasticity in this association. It provides a potential pathway to improve associative memory in older adults.

**Supplementary Information:**

The online version contains supplementary material available at 10.1186/s41235-025-00688-5.

## Introduction

Associative memory is the cognitive ability to link components together either directly or through spatial, temporal or other types of relationships (Raaijmakers & Shiffrin, 1981). Deficits in associative memory are often found with advancing age (Naveh-Benjamin, 2000; Naveh-Benjamin et al., 2003, 2004; Old & Naveh-Benjamin, 2008). There have been many studies showing age-related impairments in associative memory in later life, such as misremembering where a person was previously seen (Chen & Naveh-Benjamin, 2012) and forgetting the source of information (Boywitt et al., 2012). Many studies have attempted to understand why these associative memory errors occur, including studies appealing to the demands of attention in encoding and retrieving items and associative representations (e.g., Castel & Craik, 2003; Naveh-Benjamin et al., 2004) or those focusing on effects of encoding strategies on age differences in associative memory (e.g., Naveh-Benjamin et al.,^2003^; Dunlosky & Hertzog, 2001). However, few studies have focused on the consequences of deficits in inhibitory function. Inhibitory functioning is the ability of an individual to inhibit impulsive responses and the interference of extraneous information in order to perform relevant behaviors consistent with their intended goals (Diamond, 2013; Hasher & Zacks, 1988). One possible reason for associative deficits in older adults is that, due to a decline in inhibitory control, they may have difficulty suppressing irrelevant thoughts and actions related to the targeted association, thereby disrupting more associative memory binding (Anderson & Levy, 2007; Hasher, 2015; Hasher & Zacks, 1988; Lustig et al., 2007; Xu et al., 2024). A recent review on hyper-binding also supported this possible reason for associative memory decline in older adults, suggesting that older adults form too many untargeted associations due to a decline of attentional control (Campbell & Davis, 2024).

The properties of interfering stimuli may modulate the formation of associative representations. Interfering stimuli that are semantically related and share a gist have a greater impact on the formation of associative memories than those that are unrelated stimuli (Bürki et al., 2020; Connelly & Hasher, 1991; Hamilton & Martin, 2007). To take a real-world example, when remembering a friend’s birthday (e.g., 10 May), interference from another friend’s birthday on a nearby date (e.g., 12 May) shares the same gist and increases confusion. However, unrelated interference, such as a work deadline, would have a minimal effect on memory accuracy. However, it is not clear whether there is an age difference in this interference effect. In particular, it remains unclear whether older adults can inhibit interference with gist representations arising from multiple target associations. The fuzzy trace theory posits that episodic memories are encoded by two separate traces: a verbatim trace, which captures the specific details of an episode, and a gist trace, which captures meaning or semantic features (Brainerd & Reyna, 1990, 2004; Reyna, 1995; Reyna & Brainerd, 1995). Gist is an internal, abstract representation of meaning. Regarding the effects of aging on memory, the fuzzy trace theory asserts that older adults show deficits in verbatim representations, but their gist representations remain well preserved (Brainerd & Reyna, 2015). Recent studies have provided converging evidence that age-related deficits in associative memory are limited to the retrieval of specific representations, whether item (Abadie et al., 2021) or associative memory (Greene & Naveh-Benjamin, 2020) is tested, whereas gist representations are more likely to be retained.

In the present study, in Experiment 1, we manipulated the properties of interfering stimuli to directly examine the age differences in the effects of interference on associative memory. Specifically, based on an associative memory paradigm involving verbatim and gist representations (Greene & Naveh-Benjamin, 2022a, 2022b), interfering words are presented in gist-related and non-gist-related conditions. One possible scenario is that older adults have limited cognitive resources, such as limited working memory (Reinhart & Nguyen, 2019), leaving no room for processing of the semantic information of interfering stimuli, which is likely to result in no differences when the interfering stimuli are either gist-related or non-gist-related (Lavie, 2010). Alternatively, as the formation of gist representations is automated and independent of the formation of verbatim representations (Greene & Naveh-Benjamin, 2023a, 2023b), older adults may show a greater interference effect of gist-related stimuli compared to younger adults due to their impaired inhibitory control. In addition, we tested participants’ inhibitory function using a modified Stroop task and used conditional processing analysis to examine the role of inhibitory function in the relationship between age and associative memory performance.

In addition, we examined whether improvements in inhibitory control might be beneficial for older adults’ associative memory. Accumulating empirical evidence suggests that the targeted inhibitory function training holds promise for improving memory performance in older adults (Aizpurua & Koutstaal, 2010; Bomyea & Amir, 2011; Chiu et al., 2018; Ji et al., 2016; Nguyen et al., 2019; Xu et al., 2023). However, it remains uncertain whether and how acute inhibitory function training could ameliorate the detrimental effects of inhibitory deficits on the gist and verbatim representations of associative memory in older adults. Experiment 2 was designed to systematically examine the effects of acute inhibitory training on associative memory in older adults. The Flanker task (Eriksen & Eriksen, 1974) was selected for inhibitory training because it effectively captures interference inhibition, a process closely aligned with the cognitive demands of our experimental task. Furthermore, prior research has demonstrated the Flanker task’s robust training effects on inhibitory plasticity (Melara et al., 2018; Nguyen et al., 2019; Zhao et al., 2018). Eye-tracking was used to record eye movement indices during memory encoding with the aim of capturing the attentional distribution during the encoding phase of older adults’ memories, which may reflect the training effects of inhibitory function. In addition, structural equation modelling was used to examine the role of acute inhibitory training on older adults’ associative memory.

In summary, in two experiments of the present study, we asked four questions (see Table 1 for details) that: (1) Does gist interfere more than non-gist interference with associative memory in older adults? (2) How does inhibitory function affect the relationship between age and associative memory? (3) Does acute inhibitory training improve the associative memory in older adults? (4) How does acute inhibitory function training improve the associative memory of older adults? We employed the modified simplified-conjoint-recognition (SCR) paradigm to compare gist-related and non-gist-related interfering stimuli on associative memory. The multinomial processing tree (MPT) model was used to measure the verbatim and gist representations of associative memory (Stahl & Klauer, 2008). In Experiment 2, we used eye-tracking to measure the benefits of acute inhibitory training on inhibition. Conditional process analysis and structural equation modelling were also used to analyze the potential pathways between inhibitory deficits and acute training effects on associative memory.

**Table 1:** Design table

Experiment No	Questions	Hypotheses	Analyses	Interpretation given to outcomes
Experiment 1	1. Does gist-related interference interfere more than non-gist interference with associative memory in older adults?	(H1a) There will be no significant difference between the gist and non-gist inference conditions in both age groups. (H1b) The interference effect of the gist inference condition will be stronger for older adults than for younger adults	We will run MPT models in younger and older adults separately and analyze the significant differences between gist and non-gist interference conditions	Results are consistent with the H1a if model parameters for which the 95% CI of the condition difference estimate included 0. Results are consistent with the H1b if older adults show more gist memory under the gist-related interference condition
	How does inhibitory function affect the relationship between age and associative memory?	(H2) Inhibitory function will mediate the relationship between age and associative memory	We will conduct a conditional process analysis using PROCESS in SPSS, while controlling for years of education as a covariate	The results are consistent with the hypothesis if any indirect pathway effect that includes inhibitory function as a mediator is statistically significant (*p* < 0.05)
Experiment 2	Does acute inhibitory function training improve associative memory in older adults?	(H3) Acute inhibitory function training will improve associative memory in older adults	We will perform t-tests on associative memory accuracy between the acute training and control groups	The results are consistent with the hypothesis if the accuracy in the acute training group is significantly greater than that in the control group (*p* < 0.05)
	How does acute inhibitory function training improve associative memory in older adults?	(H4) Acute inhibitory function training will reduce the effect of gist-related interference and then improve older adults’ associative memory	We will use structural equation modeling to examine the relationships between variables, including groups, eye movement parameters, R-I, and ACC. Specifically, a latent variable was constructed using four significant eye movement parameters in the interference word AOI as proxies	The results are consistent with the hypothesis if any indirect pathway effect that includes the eye-tracking latent variable as a mediator is statistically significant (*p* < 0.05)

MPT model = multinomial-processing-tree model; 95% CI = 95% credible interval; R-I = the proportion of “Intact” response in “Related” probes; ACC = the accuracy of associative memory task; AOI = area of interest in eye movement parameters

## Experiment 1

### Methods

*Transparency and openness****.*** All procedures of Experiments 1 and 2 were approved by the Institutional Review Board (IRB) of the Faculty of Psychology, Southwest University (IRB number H22113). To support transparency and openness, all data, materials, and analysis scripts are available at OSF: https://osf.io/793ge/. Experiment 1 was pre-registered at Aspredicted:https://aspredicted.org/tq9uq.pdf . Experiment 2 was pre-registered at https://aspredicted.org/5hc9a.pdf. We also reported how we determined our sample size, any data exclusions, all manipulations, and all measures.

*Participants***.** Eighty healthy younger and older adults were recruited from Southwest University and the local community, 40 per age group respectively. The sample size was determined based on previous studies (e.g., Greene & Naveh-Benjamin, 2020), which suggested that a sample size of 30 participants per age group was sufficient to capture age-related differences in verbatim and gist memory. Participants were native Chinese speakers with normal or corrected-to-normal vision and hearing, and free of neurological and psychiatric disorders. Older adults with a score above 25 on the Mini-Mental State Examination (MMSE; Folstein et al., 1975) were included in the experiment (MMSE range: 26–30; *M* = 28.98, *SD* = 1.11). Data from one older adult was excluded because she performed below the guessing level on the memory task (ID: 13; Accuracy, ACC = 0.30). As a result, 39 older adults (33 female; age range: 57–78 years; *M* = 69.10, *SD* = 5.20; years of education: *M* = 10.15, *SD* = 2.29) and 40 younger adults (33 female; age range: 18–27 years; *M* = 21.6, *SD* = 2.25; years of education: *M* = 15.68, *SD* = 2.14) were included for subsequent statistical analyses. The timeframe of data collection was from 2022 to 2023.

*Stimuli*. The stimuli consisted of 120 scene pictures, 120 target words, and 60 interfering words, which were combined to form 120 scene-word pairs. These 120 pairs were then divided into six experimental blocks and two practice blocks. Each experimental block consisted of 16 scene-words pairs for both the study and test phases, totaling 96 pairs in each phase. The remaining 24 pairs were allocated to the practice blocks, with 12 scene-word pairs per practice block.

The scene pictures were drawn from a pool of pictures in different categories (Konkle et al., 2010). Within each category, two pictures were randomly selected. Target words were taken from the original Deese-Roediger-McDermott (DRM) list (Deese, 1959; Roediger & McDermott, 1995). Interfering words included two types: gist and non-gist. Gist interfering words were selected from DRM lists as lures that shared semantic correlations with the target words (e.g., “sleep” as the gist word for “nap” and “yawn”). Non-gist interfering words, conversely, were new words with no semantic association to the target words (e.g., “stone” as the non-gist interfering word for “school” and “schoolbag”). Prior to the experiment, we recruited 30 older and 30 younger adults to complete a materials assessment in which the participants were asked to rate on a scale of 1 to 7 how closely the two target words were semantically related to the gist word. Results showed that gist pairs (two target words and one gist word) had a higher score (*M* = 6.55, *SD* = 0.34) than non-gist pairs (two target words and one non-gist word; *M* = 1.26, *SD* = 0.33; *t*_*59*_ = 62.44, *p* < 0.001).

**Fig. 1:**
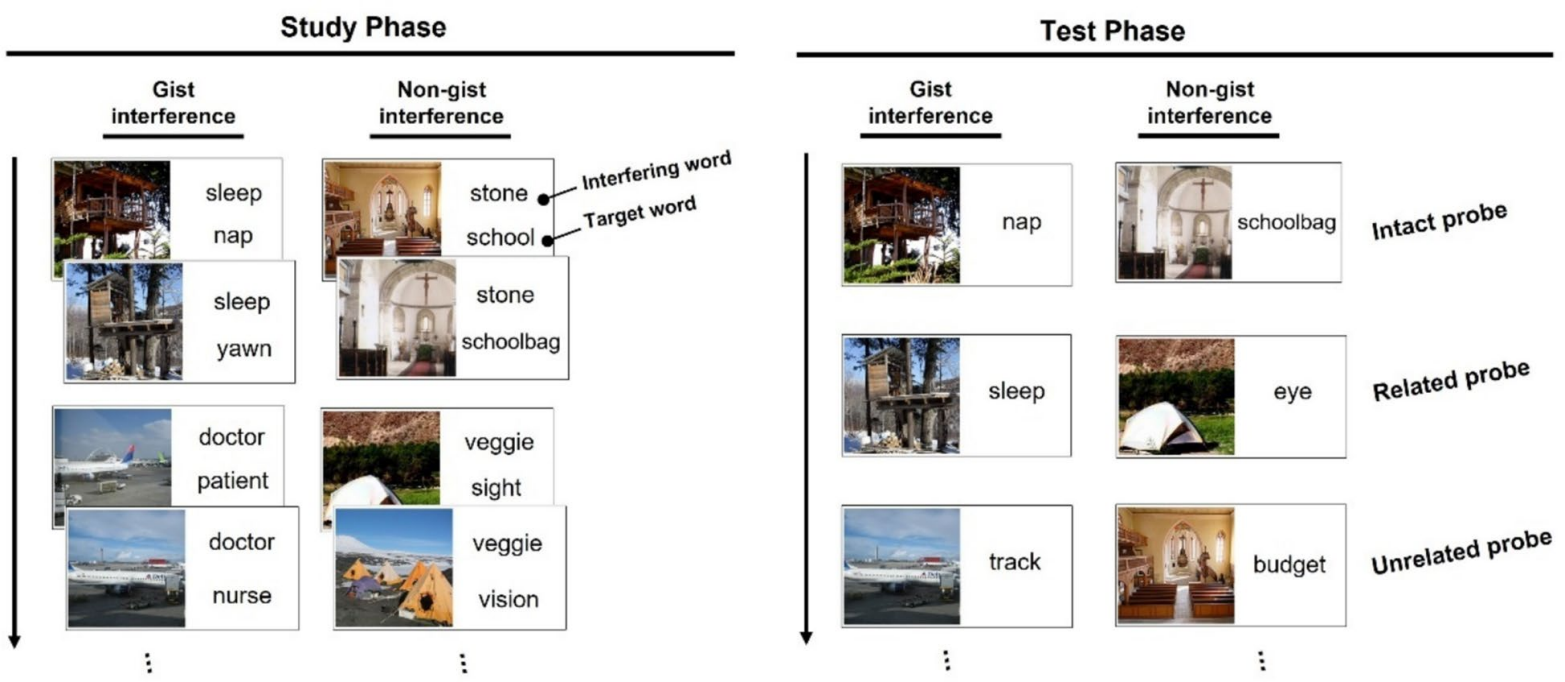
Schematic of the procedure in Experiment 1. In the study phase, participants were instructed to memorize the pairs of scene pictures and the target words at the bottom and to ignore the interfering words at the top (positions counterbalanced across subjects). Every two consecutive pairs formed a gist word memory. Interfering words consisted of gist words (i.e., gist interference) and non-gist words (i.e., non-gist interference). In the test phase, participants were instructed to decide whether the given picture-word pair was “intact”, “related”, or “unrelated”. Intact probes are picture-word pairs that have been presented previously. Related probes are pairs of scene pictures with the gist words of the original target words (e.g., “sleep” serving as one of the gist words of “nap” and “yawn”, “eye” serving as the gist words of “sight” and “vision”), and unrelated probes are pairs of scene pictures with new, non-gist words which are not semantically related to the original target words

As shown in Fig. 1, during the study phase, each stimulus included three elements: a scene picture, a target word, and an interfering word. Each scene picture was paired with a target word and an interfering word. The scene pictures in every two consecutive trials were from the same scene category, (e.g., two different treehouse pictures; see Fig. 1). Scene categories were not repeated across trials. The target words paired with scene pictures from the same category were drawn from the same DRM list (e.g., “nap” and “yawn” from one DRM list). This setup meant that two successive scene-target word pairs were supposed to form a gist associative memory, such as “treehouse-sleep.” The interfering words were used to create either a gist interference condition (e.g., sleep) or a non-gist interference condition (e.g., “tent-eye” association with “veggie” as the inferring word). Every two consecutive interfering words were the same in the study phase.

During the test phase, three distinct types of probes were used: intact, related, and unrelated. All three probe types were derived from both the gist interference and non-gist interference conditions presented during the study phase. Intact probes were pairs of scene pictures with the original target words previously presented in the study phase, for example, “tree house picture 1” paired with “nap”. Related probes were pairs of scene pictures with the gist words of the target words. For example, in the gist interference condition, “tree house picture 2” was paired with “sleep”, which served as the gist word of “nap” and “yawn”. Conversely, in the non-gist interference condition, “tent picture 1” was paired with “eye” (the gist word for “sight” and “vision”) for the related probes. Unrelated probes were pairs of scene pictures with new, non-gist words that were not semantically related to the original target words—for example, “plane picture 2” paired with “track”, where “track” was unrelated to the studied target words “patient” and “nurse”. The scene-word pairs in the test phase differed from those in the study phase in that each scene was paired with a single word (see Fig. 1). Each test block consisted of five intact probes, six related probes, and five unrelated probes.

*Procedure*. Stimulus pairs were presented using E-prime 2.0 software (Schneider et al., 2012), and the experiments were conducted in a standardized behavioral laboratory at Southwest University. Each participant completed the experiment individually in the presence of an experimenter in the laboratory. The experiment lasted approximately one hour. Each block consisted of a study phase and a test phase (see Fig. 1). During the study phase, each scene-words pair was pseudorandomly presented on the screen for four s, followed by a blank screen for 0.3–0.8 s to prevent anticipation. Participants were instructed to remember the picture and the word at the bottom and to ignore the interfering word at the top. The positions of the two types of words on the screen were counterbalanced across participants. Participants were informed that there were no repeated pictures or target words. Sixteen scene-words pairs in a block were presented pseudorandomly, with the constraint that two pairs eliciting the same gist were presented consecutively in the same block. The study phase of each block was followed by a 5-s interval, after which the test phase began.

During the test phase, participants were instructed to judge whether the scene-word pairs presented on the screen had been studied in the previous phases. If so, they were instructed to press “a” (“intact probe”) on the keyboard. Otherwise, they were asked to judge whether the new paired word had a semantic relationship with the old paired word (i.e., the target word). If yes, they were instructed to press “f” (“related probe”); if no, they were instructed to press “j” (“unrelated probe”). There was no time limit for responding. Due to known age differences in learning, we adjusted the task difficulty for different age groups (Sander et al., 2021). Specifically, older adults were tested after every block they learned, whereas younger adults were tested after every two blocks.

Two minutes after the memory test, participants were asked to complete a Stroop task (Stroop, 1935) to assess their inhibitory function. In the modified Stroop task, participants were instructed to judge whether the color of the word matched the meaning of the word. For example, the Chinese character “红” means “red”, but it is yellow, so the color of the word does not match its meaning. Participants were to press the “d” key if the color and meaning matched and the “h” key if they did not. The task consisted of 12 practice trials and 80 formal trials, including 40 consistent trials and 40 inconsistent trials.

### Data analyses

*Response proportion****.*** For each probe (i.e., intact, related, and unrelated), the proportions of responses under different interference conditions (gist interference vs. non-gist interference) were calculated separately for older and younger adults. ANCOVA was then used to examine the age by condition interaction effect for correct and incorrect responses in each probe, with education included as a covariate.

**Fig. 2:**
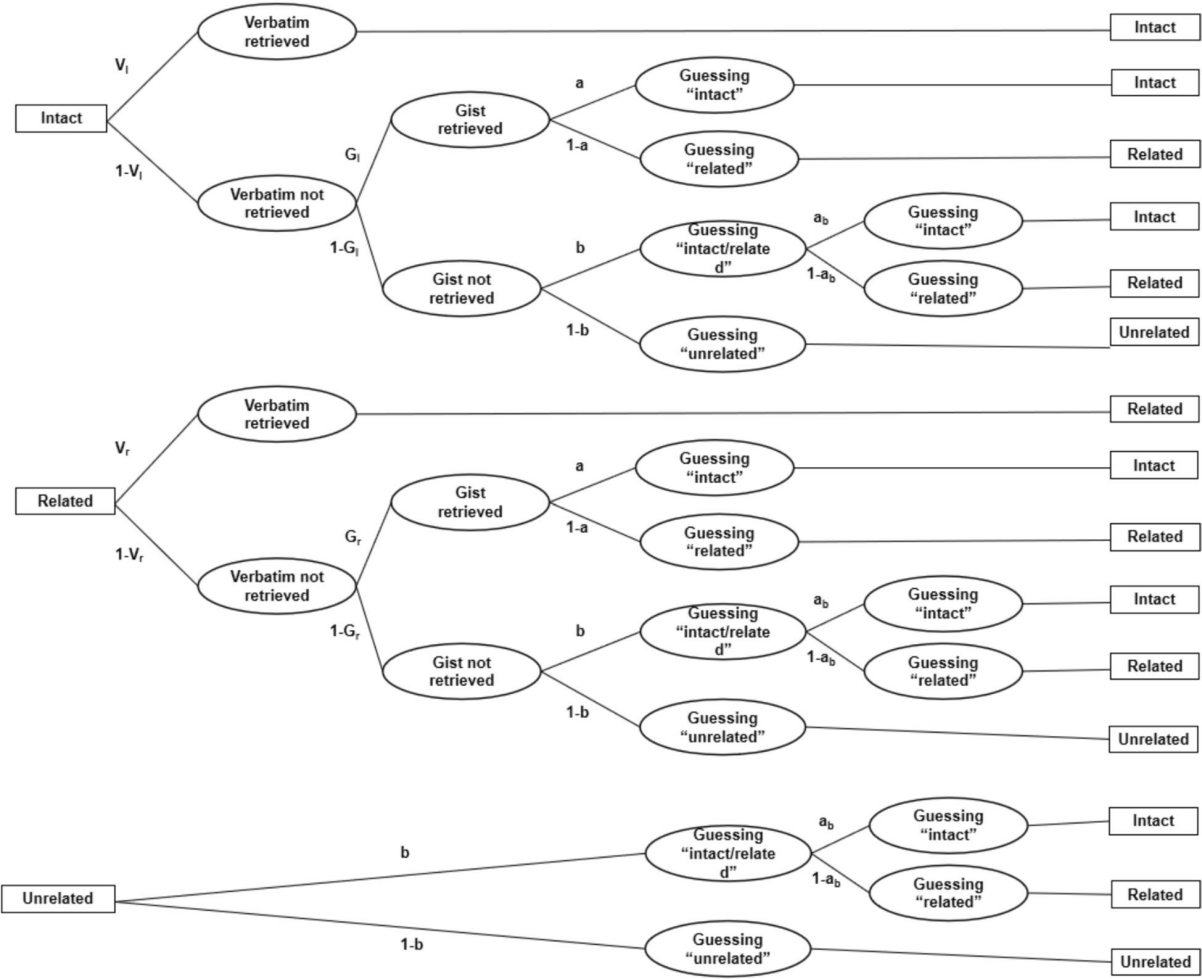
The Multinomial-processing-tree(MPT) model. The model picture shows how people make their responses (boxes on the right) to a given test probe (i.e., intact, related, and unrelated; boxes on the left) based on the constructed different cognitive processes (ovals). The probabilities of the different cognitive processes are represented by the parameters on the paths. Two V parameters refer to the probabilities of retrieving verbatim memory for intact (Vi) or related (Vr) probes, and two G parameters refer to the probabilities of retrieving gist memory for intact (Gi) or related (Gr) probes. In gist retrieval states, individuals must guess whether the probe is intact (a) or related (1-a). If they retrieve neither verbatim nor gist memory, they still guess whether the probe is intact with probabilities b and 1-b. In this situation, people make a final guess of intact with a probability of ab, or related with a probability of 1—ab

*MPT model.* The multinomial-processing-tree (MPT) model (Klauer, 2010) was utilized to differentiate and quantify verbatim and gist memory, which is reflected by the corresponding parameters V and G, as shown in Fig. 2. It models gist retrieval conditional on the participant failing to remember the specific/verbatim representation of the studied item. The MPT model provides a plausible and unique modelling interpretation of the responses made by the subjects under different experimental probe conditions. An attempt is made to isolate the process of access to verbatim and gist trace in the subjects’ responses by the paths (i.e. oval) taken to reach the different responses and the corresponding parameters (i.e. probabilities) based on the fuzzy trace theory.

We applied both Greene’s MPT model (Greene & Naveh-Benjamin, 2020) and original MPT model (Stahl & Klauer, 2008) to data from younger and older adults under both interference conditions. Greene’s MPT model showed a better fit (see Figure S1 and posterior predictive *p*-value in Supplemental Material) and was therefore used for all subsequent MPT analyses. Greene’s model assumed that participants’ bias to respond “intact” can change depending on whether the gist is retrieved, while the original SCR paradigm (Stahl & Klauer, 2008) included an “a” parameter for responding “intact”, regardless of gist. We used the *TreeBUGS* package in *R* (Heck et al., 2018; Team, 2013) to run the model separately in both age groups to compare the differences between the gist and non-gist interference conditions. These models were fitted as hierarchical Bayesian models and education was controlled as a covariate (Kruschke, 2011, 2018). Forest plots were then used to visually display the interference condition differences by subtracting the estimates of non-gist interference from those of gist interference in younger and older adults, respectively (Abadie et al., 2021). Parameters for which the 95% CI of the difference estimate included 0 indicated that a credible effect was not detected (Smith & Batchelder, 2010). Additional information on the MPT model (e.g., number of MCMC chains and iterations) can be found in the Supplemental Material. All the MCMC chains converged on stable posterior distributions for each parameter (R-hat values < 1.05).

*Inhibitory Function Index (IF) from Stroop task****.*** The response time (RT) in the consistent condition was subtracted from the RT in the inconsistent condition and then divided by the RT in the consistent condition. For ease of interpretation, the above score was subtracted from a larger number (i.e., two). Thus, the higher the score, the better the inhibitory function. In addition, to account for the trade-off between accuracy and speed, the reaction time was multiplied by the accuracy. Therefore, the final equation is as follows:

$${\text{IF}} = {\text{ACC*}}\left[ {2 - \frac{{RT_{inconsistent} { } - { }RT_{consistent} }}{{RT_{consistent} }}} \right]$$

*Conditional process analysis.* To explore the underlying relationship between age, inhibitory function, and memory performance, a conditional process analysis (Hayes & Rockwood, 2020) was conducted using PROCESS in SPSS (http://www.afhayes.com), while controlling for years of education as a covariate.

### Results

**Fig. 3:**
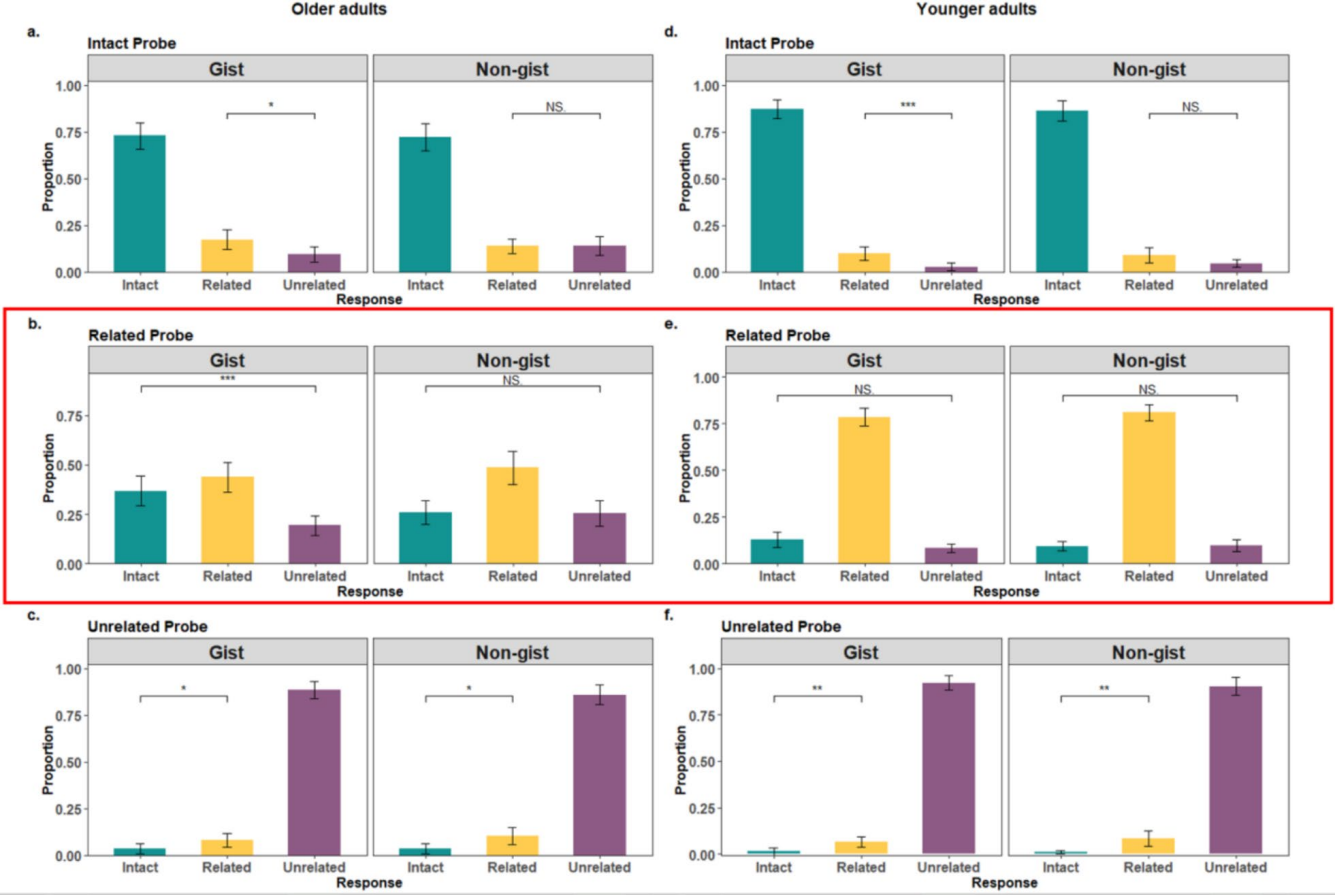
Response proportions of older adults (a, b, c) and younger adults (d, e, f) in the gist interference and non-gist interference in three probe types (intact, related, and unrelated probe). The red box shows the interaction effect of age and interference condition on the proportion of related incorrectly identified as intact, R-I. Note: Significance reflects the contrast of incorrect responses for each probe (e.g., “related” versus “unrelated” responses to Intact probes). *p < 0.05; **p < 0.01; ***p < 0.001, NS = not significant

*Response proportion****.*** The response proportions of older and younger adults in the gist and non-gist interference conditions for three probe types (intact, related, and unrelated probe) are shown in Fig. 3. For accuracy (i.e., participants responded “intact” to intact probes, “related” to related probes, and “unrelated” to unrelated probes), a 2 (group: younger, older) × 2 (interfering condition: gist, no-gist) × 3 (probe: intact, related, unrelated) mixed-design ANCOVA was conducted by *afex* in *R* (https://github.com/singmann/afex), while education served as a covariate. Group had a significant main effect on accuracy (*F*_(1, 76)_ = 38.85, *p* < 0.001, *η*^*2*^ = 0.34), with older adults (*M* = 0.69, *SD* = 0.27) performing significantly worse than younger adults (*M* = 0.86, *SD* = 0.15). Probe also showed a significant main effect on accuracy (*F* = 82.99, *p* < 0.001, *η2* = 0.52). Specifically, related probes (*M* = 0.63, *SD* = 0.26) led to significantly worse accuracy compared to both intact probes (*M* = 0.80, *SD* = 0.20) and unrelated probes (*M* = 0.89, *SD* = 0.15). In contrast, the main effect of interfering condition was not statistically significant (*F* = 0.04, *p* = 0.85, *η*^*2*^ < 0.001). Importantly, the group x probe interaction was statistically significant (*F*_(2,152)_ = 26.09, *p* < 0.001, *η*^*2*^ = 0.25). Post-hoc pairwise comparisons (Tukey-adjusted) revealed that across probe types, older adults performed significantly worse (*M* = 0.50, *95%CI* = [0.43, 0.58]) than younger adults (*M* = 0.76, *95%CI* = [0.69, 0.83]) on related probes (*t* ratio_*(76)*_ = -4.02, *p* < 0.001). In contrast, no significant age differences were observed for intact probes (*t* ratio_*(76)*_ = -1.53, *p* = 0.13) or unrelated probes (*t* ratio_*(76)*_ = -0.81, *p* = 0.41). No other two-way or three-way interactions reached statistical significance (*ps* > 0.05).

To explore the potential behavioral mechanisms accounting for the accuracy differences between the two age groups, we analyzed the incorrect responses for each group, as shown in Fig. 3. To avoid numerous enumeration analyses, we took a two-step analytic approach. First, we analyzed the patterns of incorrect responses in older and younger adults with the aim of exploring the potential age-related differences. Second, we focused on the target incorrect responses to confirm whether age-related differences existed. For pattern analyses as shown in Fig. 3, older adults exhibited similar error patterns to younger adults for intact and unrelated probes, indicating that the proportion of incorrect "related" responses was significantly higher than the proportion of other responses (for older adults, intact probes: *t*_*72*_ = 2.48; *p* = 0.01, Cohen’s *d* = 0.56; unrelated probes: *t*_*70*_ = 2.15; *p* = 0.03, Cohen’s *d* = 0.49; for younger adults, intact probes:*t*_*62*_ = 3.46; *p* < 0.001, Cohen’s *d* = 0.78; unrelated probes: *t*_*62*_ = 5.48; *p* = 0.001, Cohen’s *d* = 0.78). Importantly, there were clearly different patterns between older and younger adults for the related probes, especially in the gist interference condition. Older adults showed a significantly higher proportion of incorrect “intact” responses (R-I) than incorrect “unrelated” responses (R-U) for the related probes under the gist interference condition (*t*_*65*_ = 3.94; *p* < 0.001, Cohen’s *d* = 0.89), whereas younger adults did not show this pattern (*t*_*62*_ = 1.97; *p* = 0.05, Cohen’s *d* = 0.44), which indicates potential age-related differences. We ran group x interference condition ANCOVA on “Intact” response in related probes and “Unrelated” response in related probes, separately, while education was controlled to explore age-related differences. The results showed that for related probes, there was a significant interaction effect of between age group and interference condition on the proportion of “Intact” response in related probes (R-I; *F*_(1,76)_ = 3.98, *p* = 0.04, *η*^*2*^ = 0.11). Post-hoc pairwise comparisons (Tukey-adjusted) revealed that under the gist interference condition, older adults had a significantly higher proportion of “Intact” response in related probes than the non-gist interference condition (*t* ratio_*(76)*_ = 3.92, *p* < 0.001), whereas for younger adults, there was no significant difference in RI between the two interference conditions (*t* ratio_*(76)*_ = 1.33, *p* = 0.19). In contrast, there was no significant interaction effect for “Unrelated” response in related probes (R-U; *F*_(1,76)_ = 1.49, *p* = 0.22, *η*^*2*^ = 0.02). These results suggest that older adults were more likely to mistake a related probe as “intact” after seeing a related distracting word at encoding, while younger adults were better at identifying related probes overall and unaffected by the relatedness of distractors at encoding.

In summary, analysis of correct responses (i.e., accuracy) revealed a statistically significant age-related differences, with older adults performing worse than younger adults. Further analysis of incorrect responses explored potential reasons for the age-related difference in accuracy, revealing the core role of “Intact” response in related probes, which was subsequently used in the mediation model analysis.

**Fig. 4:**
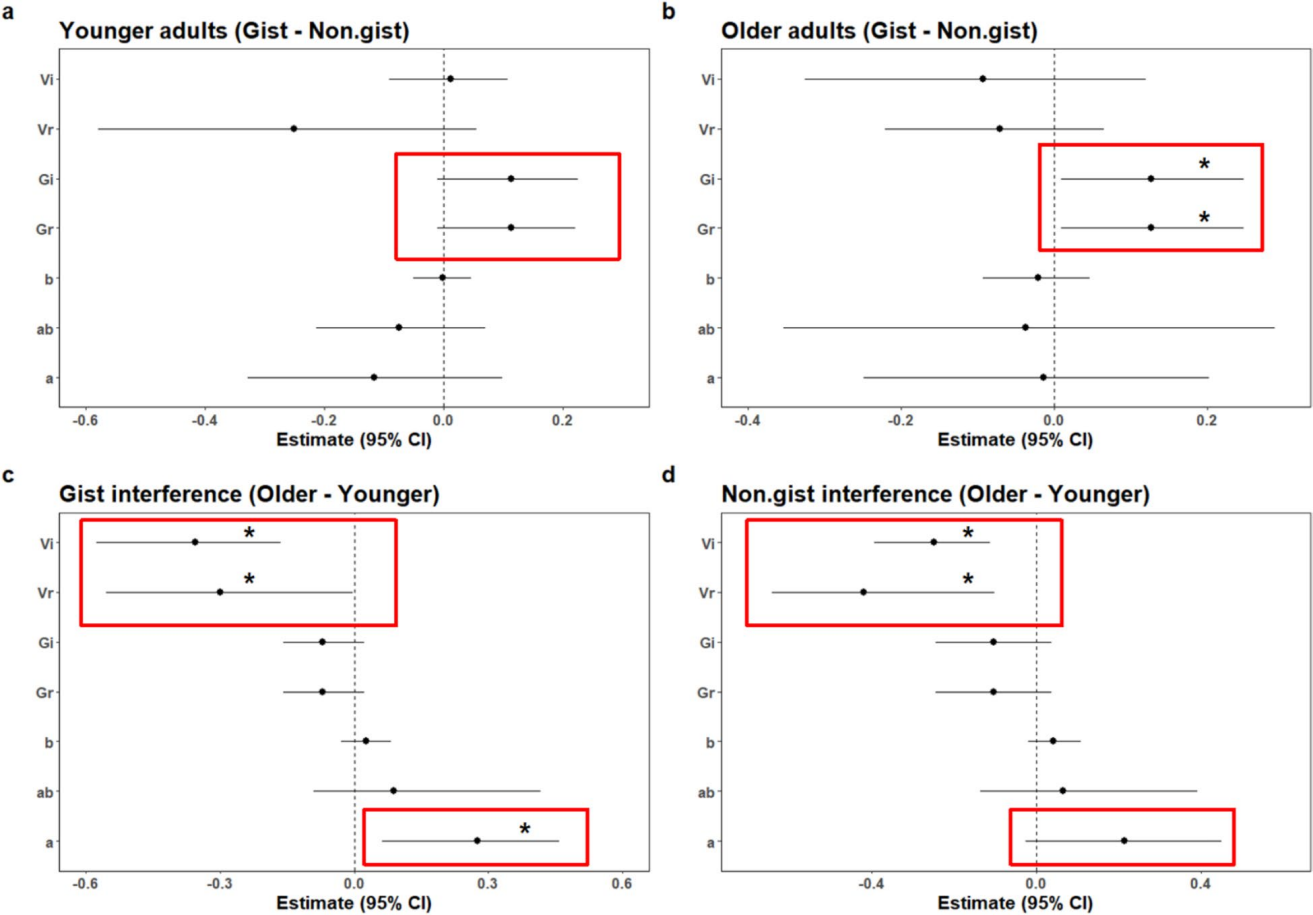
Forest plots show the differences in the interference conditions (gist minus non-gist) and age groups (older minus younger) for each parameter in (a) younger adults; (b) older adults; (c) gist interference condition; (d) non-gist interference condition. Red boxes show the age-related difference in model parameters. Vi = verbatim trace is retrieved given an intact probe; Vr = verbatim trace is retrieved given a related probe; Gi = gist trace is retrieved given an intact probe; Gr = gist trace is retrieved given a related probe; a = guessing “intact” on retrieving the gist; ab = guessing “intact” on entering state b; b = guessing “intact” or “related” when gist is not retrieved

*MPT results.* We ran four separate MPT models among two age groups and two interference conditions while controlling education as a covariate. The details of the parameter estimate for each interference condition among younger and older adults were shown in Table 2. Figure 4a, b show the interference differences for each parameter obtained by subtracting the non-gist from the gist condition in younger and older adults, respectively. There were no significant differences in the parameters between conditions in younger adults (Fig. 4a), while older adults showed significantly higher values of *G*_*i*_ and *G*_*r*_ in the gist interference condition compared to the non-gist condition (Fig. 4b). Figure S2 (see Supplemental Materials) shows the posterior probability distribution of *Gi* for older and younger adults in the gist and non-gist interference conditions, which directly reflects the different distribution patterns of gist and non-gist interference conditions across the two age groups. Although Fig. 4 shows remarkably similar directional patterns of gist interference effects on parameter *G* across the two age groups, the present results (Figure S2) indicate the true differences of *G* between gist and non-gist interference conditions in older adults. Figures 4c, d show the age differences for each parameter obtained by subtracting the younger adults from the older adults in gist and non-gist interference conditions, respectively. The results showed that, compared to younger adults, older adults exhibited significantly lower verbatim estimate values (*V*_*i*_ and *V*_*r*_) in both interference conditions. In terms of the guessing parameter *a*, older adults showed significantly higher values in the gist interference condition than younger adults, though there was no significant difference in the non-gist condition.

**Table 2:** MPT Parameter Estimates for interference condition in Experiment 1

Parameters	Younger adults		Older adults	
	Gist	Non-gist	Gist	Non-gist
Vi	.87 [.79, .93]	.86 [.78, .92]	.51 [.30, .68]	.61 [.48, .73]
Vr	.33 [.04, .58]	.52 [.23, .71]	.03 [.00, .11]	.11 [.01, .25]
Gi = Gr	.85 [.78, .91]	.76 [.66, .86]	.78 [.72, .84]	.66 [.56, .76]
a	.23 [.12, .40]	.31 [.17, .49]	.51 [.38, .65]	.53 [.36, .72]
ab	.04 [.00, .15]	.09 [.01, .21]	.13 [.01, .45]	.16 [.01, .47]
b	.05 [.02, .09]	.05 [.02, .10]	.08 [.04, .13]	.10 [.05, .15]

Estimates are group-level means; values in brackets are 95% Bayesian credible intervals. Vi = verbatim trace is retrieved given an intact probe; Vr = verbatim trace is retrieved given a related probe; Gi = gist trace is retrieved given an intact probe; Gr = gist trace is retrieved given a related probe; a = guessing “intact” on retrieving the gist; ab = guessing “intact” on entering state b; b = guessing “intact” or “related” when gist is not retrieved

We extracted estimates of verbatim (*V*_*i*_ and *V*_r_) and gist memory (*G*_*i*_) from the MPT model results for each participant. The average of *V*_*i*_ and *V*_*r*_ (*V*_*m*_) was used to represent an individual’s verbatim memory. Since Greene’s model imposes a constraint that *G*_*i*_ equals *G*_*r*_, only *G*_*i*_ was retained as the gist memory indicator. The verbatim and gist memory estimates provided other representative estimates about associative memory performance more than accuracy. These verbatim and gist memory estimates were used in later correlation and model analyses.

In summary, based on various parameter estimates, MPT models provided a more nuanced understanding of the differences across interference conditions and age groups than accuracy measures alone. The results indicate that older adults exhibited more gist memory (*G*_*i*_ and *G*_*r*_) in the gist interference condition than in the non-gist condition, a pattern not observed in younger adults. When comparing age groups, both the gist and non-gist interference conditions showed that older adults exhibited less verbatim memory (*V*_*i*_ and *V*_*r*_) than younger adults. However, only the gist interference condition revealed that older adults guessed “intact” (*a*) more often. These verbatim- and gist-related model estimates were subsequently used in correlation and mediation model analyses to represent different facets of associative memory performance.

*Inhibitory function.* The Stroop results showed that the inhibitory function of older adults (*M* = 1.73; *SD* = 0.22) was significantly worse than that of younger adults (*M* = 1.87; *SD* = 0.12; *t*_*78*_ = 3.61, *p* < 0.001, Cohen’s *d* = 0.82). Additional exploratory analyses were conducted to examine the correlations between inhibitory function, memory performance, and response proportion. The results show that after FDR correction for multiple comparisons (Benjamini & Hochberg, 1995), inhibitory function was positively correlated with memory task accuracy (ACC; *r* = 0.23, *p* = 0.004), verbatim memory (*V*_m_; *r* = 0.23, *p* = 0.003), whereas negatively correlated with “Intact” response in related probes (*r* = −0.35, *p* < 0.001).

**Fig. 5:**
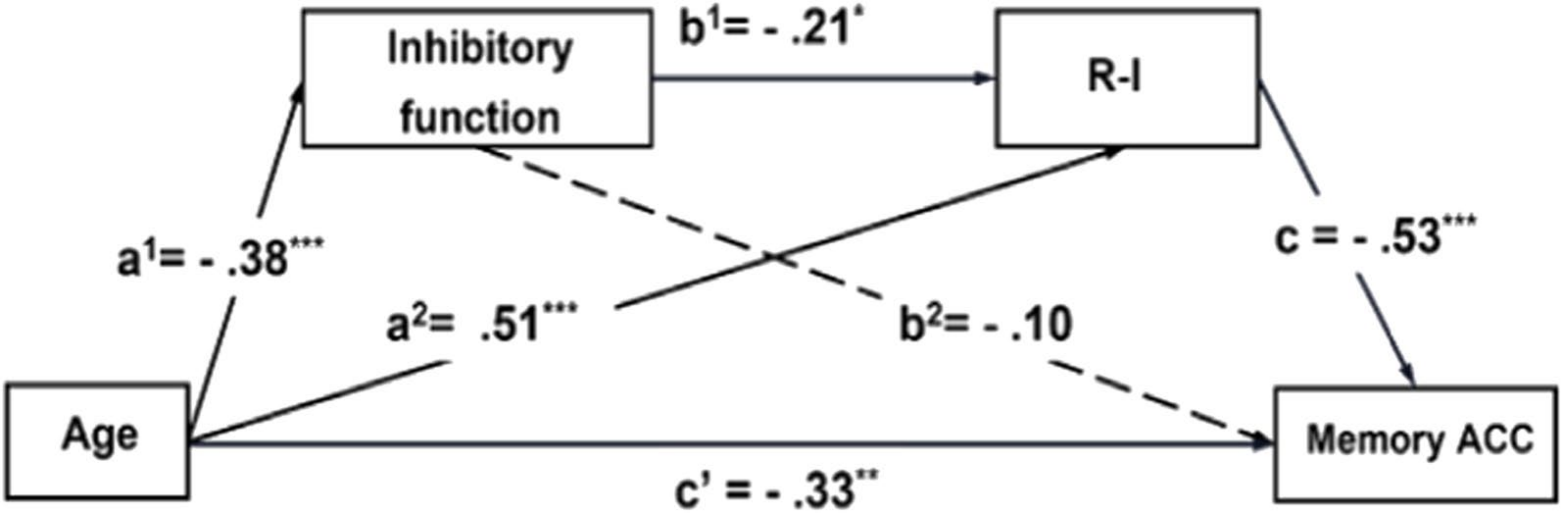
Chain mediation models. Age affected memory ACC through a chain mediation pathway in which inhibitory function and R-I acted as mediators. After accounting for the mediating effects of inhibition and R-I, the residual effect of age on memory ACC was still statistically significant (*c’* = -0.33, *p* < 0.01). Age = younger (1) and older adult (2); R-I = the proportion of “Intact” response in related probes; ACC = the accuracy of associative memory task. *p < 0.05; **p < 0.01; ***p < 0.001

*Mediation model.* We conducted exploratory analyses using chain mediation models separately for each age group to examine the relationships between inhibitory function and memory performance (ACC, Vm, and Gi), as well as the relationships between age groups and incorrect responses. The models were run using PROCESS in SPSS (Model 6) while controlling for years of education as a covariate. Table S2 (see Supplemental Material) presents the results of all mediation models. Focusing on the core role of wrong “Intact” response in related probes identified in our earlier response analysis, a statistically significant chain mediation model was revealed (see Fig. 5). Table S1 (see Supplemental Material) presents the results of the valid chain mediation model, which includes Model 1, 2, and 3, and the conditional indirect effects analysis. Model 1 tested the effects of age groups on inhibitory function, Model 2 tested the effects of age groups and inhibitory function on “Intact” response in related probes, and Model 3 tested the effects of age groups, inhibitory function, and “Intact” response in related probes on memory ACC. The conditional indirect effect analysis analyzed the effects of the mediation process.

Model 1 (*F* = 13.21, *R*^*2*^ = 0.15,* p* < 0.001), Model 2 (*F* = 23.30, *R*^*2*^ = 0.38, *p* < 0.001), and Model 3 (*F* = 28.15, *R*^*2*^ = 0.53, *p* < 0.001) showed that age groups negatively correlated with memory ACC (*β* = −0.33, *p* = 0.002). In addition, age groups negatively predicted inhibitory function (*β* = −0.38, *p* < 0.001) and positively predicted “Intact” response in related probes (*β* = 0.51, *p* < 0.001). Inhibitory function negatively predicted “Intact” response in related probes (*β* = −0.21, *p* = 0.04) and had no relationship with memory ACC (*β* = −0.10, *p* = 0.25). “Intact” response in related probes negatively predicted memory ACC (*β* = −0.53, *p* < 0.001). These results revealed a chain mediation model in which age groups affected memory ACC through a chain mediation pathway in which inhibitory function and “Intact” response in related probes acted as mediators (see Fig. 5). Detailed model results are provided in Table S1 in the Supplemental Material.

To explore the effects of interference manipulation on associative memory, we ran mediation models to examine the relationships between the interference condition, “Intact” response in related probes, and ACC separately in older and younger adults, while controlling years of education as a covariate. For older adults, the results revealed a mediation model where “Intact” response in related probes served as a mediator (*β* = − 0.04, *p* = 0.03). This indicates that older adults exhibited more inaccurate “Intact” response in related probes responses (*β* = 1.94, *p* = 0.02) in the gist interference condition, which in turn led to poorer accuracy (*β* = − 0.02, *p* < 0.001). However, younger adults did not demonstrate this chain mediation (*β* = − 0.02, *p* = 0.24).

In summary, the chain mediation model uncovered the behavioral mechanism of inhibition-related age differences in accuracy. Compared to younger adults, older adults had poorer inhibitory function, which led to an increase in incorrect “Intact” response in related probes responses and, consequently, worse associative memory accuracy. Furthermore, the mediation model for older adults concerning interference conditions highlighted the critical role of the incorrect “Intact” response in related probes in their memory performance. Specifically, gist interference exacerbated the effect of interference on older adults, causing them to exhibit more incorrect “Intact” response in related probes responses and resulting in worse accuracy.

### Discussion

In Experiment 1, we explored (1) whether gist representation interfered more than non-gist with associative memory in older adults, and (2) how inhibitory function affected the relationship between age and associative memory accuracy. We discovered that, compared to the non-gist interference condition, the gist condition had a greater interfering effect on older adults. The mediation model suggests that older adults had more incorrect “Intact” response in related probes responses in the gist interference condition, which led to decreased accuracy. Younger adults did not show this pattern. This interfering effect increased gist memory of older adults, which was supported by MPT model results, under gist interference conditions, when participants failed to retrieve the specifics of the original item, they were nonetheless more successful at retrieving its gist, compared to matched situations of non-gist interference and failing to retrieve the specifics of the original item. This enhanced gist memory may be viewed as a byproduct of impaired inhibitory control. Although gist memory increased, it reflected an inability to suppress interference from gist-related words that were meant to be ignored. This also implies that gist representation requires fewer cognitive resources for processing and integration into gist memory (Greene & Naveh-Benjamin, 2023a, 2023b).

Additionally, we also discovered that inhibitory function played a mediation role between age and associative memory accuracy. This was proved by a chain mediation model, which revealed that when age increased, inhibitory function declined, which resulted in more wrong responses specific to related probes (i.e., “Intact” response in related probes) and eventually decreased their associative memory accuracy. Besides these two main findings, age-related differences in inhibitory function index from the Stroop task suggest inhibitory deficits in older adults. The significant differences in accuracy between two age groups indicate that older adults had worse associative memory than younger adults. The only significant interaction between age and interference condition on “Intact” response in related probes suggests that older adults struggled more with gist-related interference.

Our results also highlight the crucial role of “Intact” response in related probes errors in age-related differences in associative memory performance. Specifically, “Intact” response in related probes responses were more sensitive to experimental manipulations than other incorrect responses, suggesting this may be a key contributor to older adults’ reduced accuracy. These results are in line with previous studies which show older adults have more “Intact” response in related probes error responses compared to younger adults (Abadie et al., 2021; Greene & Naveh-Benjamin, 2020). In our results, regarding related probe, older adults’ increased “Intact” response in related probes errors in the gist interference condition reflect a failure to suppress irrelevant but familiar gist information, resulting in source confusion during retrieval and a greater tendency to incorrectly response “intact”. As shown in Fig. 3, accuracy for related probes was disproportionately lower among older adults compared to the other probe types. Importantly, mediation analyses confirmed that “Intact” response in related probes, rather than other incorrect responses (e.g., R-U), mediated the relationship between age group and associative accuracy. Moreover, as shown in Fig. 4, MPT modeling further supported this interpretation by revealing an increased estimate of parameter *a* (the probability of guessing “intact” when retrieving gist) for older adults under gist interference conditions, compared to younger adults.

## Experiment 2

Experiment 1 elucidated the pathway by which inhibitory function deficits impair associative memory in older adults, and in Experiment 2 we further tested whether and how acute inhibitory training could ameliorate the detrimental effects of gist-related interference on associative memory. The Flanker task was selected as the acute training task for two main reasons. First, it is a well-established paradigm for measuring interference inhibition, which closely aligns with the cognitive processes involved in our experimental task—the inhibition of interference from irrelevant word stimuli. Second, prior research has demonstrated that the Flanker task can induce short-term improvements in inhibitory control, suggesting its potential utility as an acute training tool (e.g., Melara et al., 2018; Nguyen et al., 2019; Zhao et al., 2018). Eye-movement techniques were used to capture the attentional distribution during the encoding phase of older adults’ memories, which reflected the training effects on inhibitory function.

### Method

*Participants*. Sixty-six older adults were recruited to participate in Experiment 2. Participants were randomly assigned to either an acute inhibitory function training group or a control group. Data from three participants in the acute-training group and four participants in the control group were excluded due to eye tracking malfunction. As a result, 30 older adults (two males; age: *M* = 67.27, *SD* = 4.02; years of education: *M* = 10.23, *SD* = 2.62; MMSE: *M* = 28.93, *SD* = 1.17) in the acute-training group and 29 older adults (two males; age: *M* = 67.90, *SD* = 4.89; years of education: *M* = 11.21, *SD* = 2.66; MMSE: *M* = 28.93, *SD* = 0.92) in the control group were included in the subsequent statistical analyses. There were no significant differences in age, sex, years of education, or MMSE scores between the two groups (see Table S3 in the Supplemental Material). The timeframe of data collection was from 2022 to 2023.

*Procedure*. Older adults in the acute-training group performed the Flanker task (Eriksen & Eriksen, 1974; see the Supplementary Material for a Description of the Flanker task) prior to the memory task. Successful training was determined by an accuracy greater than 90%, otherwise additional rounds of the Flanker task were administered. Only one older adult required two rounds of training to meet the requirements, while the rest received one round. After acute-training, older adults proceeded immediately to the memory task. Older adults in the control group skipped the Flanker task and completed the memory task directly.

Participants’ eye movements were recorded throughout study phase of the memory task. The experiment was conducted in a standardized eye movement laboratory. Images were presented on a 20-inch monitor using E-Prime 2.0. The resolution of the laboratory computer screen was 1920 × 1080 pixels, with a refresh rate of 144 Hz. The distance between the screen and the subject’s eyes was approximately 65 cm. The eye movement device used was an Eyelink 1000 (SR Research Ltd., Mississauga, ON, Canada) with a sampling rate of 500 Hz. All participants recorded information from their right eye and underwent a nine-point calibration procedure followed by a nine-point validation.

### Data analyses

*Eye movements.* Three areas of interest (AOIs) were defined: figure AOI, target word AOI, and interfering word AOI. Fixations that were not in the AOI or were shorter than 100 ms were excluded. Fixation duration (FD), fixation frequency (FF), and their proportions at each AOI (FD_P and FF_P) were extracted for subsequent analyses.

*MPT model.* MPT models were also used to explore any acute-training effects on different model parameters.

*Structural Equation Modeling.* Structural equation modeling (SEM; Kline, 2023) was used to examine the relationships among variables, including groups, eye movement parameters, “Intact” response in related probes, and ACC, in order to explore the cognitive psychological pathway underlying the effect of acute inhibitory function training on associative memory. Specifically, a latent variable was constructed using four significant eye movement parameters in the interfering word AOI as proxies. The *lavaan* package in *R* 4.3.3 was used for these analyses (Rosseel, 2012). The χ^2^/df, comparative fit index (CFI), Tucker-Lewis index (TLI), standardized root mean square residual (SRMR), and root mean square error of approximation (RMSEA) were considered as model fit indices (Bentler, 1990a, 1990b). A value of χ^2^/df less than 2, a value of CFI and TLI greater than 0.90, and a value of SRMR and RMSEA less than 0.08 indicated a good model fit (Bentler, 1990a, 1990b).

### Results

**Fig. 6:**
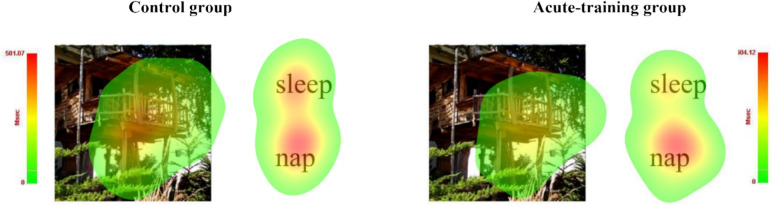
Hotspot maps of older adults’ fixation durations in the acute-training and control groups

*Eye movement results.* Table 3 shows the values and differences of the eye movement parameters of the three AOIs in the acute-training and control groups respectively. For the interfering word AOI, there were significant group differences in FD (*t*_*57*_ = − 2.30, *p* = . 025, *Cohen’s d* = − 0.60), FD_P (*t*_*57*_ = − 2.51, *p* = . 016, *Cohen’s d* = -0.66), FF (*t*_*57*_ = − 3.14, *p* = . 003, *Cohen’s d* = − 0.83), and FF_P (*t*_*57*_ = − 2.90, *p* = . 006, *Cohen’s d* = − 0.76), while there were no group differences in the target word AOI and figure AOI (*ps* > . 05). Figure 6 shows the eye movement hotspots of an exemplar stimulus in which the fixation duration of the interfering word AOI was significantly shorter in the acute-training group than in the control group. In short, the eye movement results revealed a valid acute training effect, with more attention resources distributed to the target words than to the interference words.

**Table 3:** The eye movement indices in the acute-training and control groups

AOIs	Eye movement parameters	Training group *(M* ± *SD)*	Control group *(M* ± *SD)*	*t*	*p*	Cohen’s *d*
Interfering word AOI	Fixation duration	275.92 ± 306.82	495.91 ± 416.45	− 2.30	0.025	− 0.60
	Proportion of fixation duration	0.08 ± 0.08	0.15 ± 0.13	− 2.51	0.016	− 0.66
	Fixation frequency	1.16 ± 1.06	2.42 ± 1.90	− 3.14	0.003	− 0.83
	Proportion of fixation frequency	0.08 ± 0.08	0.16 ± 0.13	− 2.90	0.006	− 0.76
Target word AOI	Fixation duration	967.47 ± 642.54	861.87 ± 612.99	0.65	0.521	0.17
	Proportion of fixation duration	0.29 ± 0.18	0.25 ± 0.17	0.86	0.396	0.23
	Fixation frequency	3.96 ± 2.30	3.55 ± 2.06	0.72	0.473	0.19
	Proportion of fixation frequency	0.29 ± 0.17	0.25 ± 0.15	0.99	0.327	0.25
Figure AOI	Fixation duration	1991.44 ± 760.12	1855.43 ± 933.34	0.61	0.542	0.16
	Proportion of fixation duration	0.60 ± 0.21	0.56 ± 0.27	0.63	0.532	0.17
	Fixation frequency	8.05 ± 2.83	7.81 ± 3.61	0.29	0.771	0.07
	Proportion of fixation frequency	0.59 ± 0.20	0.54 ± 0.25	0.81	0.421	0.22

AOI = area of interest

*Response proportion and MPT results.* Response proportion results showed no significant differences in accuracy between the acute-training and control groups (see Figure S3 for details in Supplemental Material). The MPT results also showed no significant differences in any of the MPT model parameters between the acute-training and control groups. Table 4 shows the results of the parameter estimates for each group under gist and non-gist interference conditions. Forest plots showed the comparisons between two groups (see Figure S4 for details in Supplemental Material).

**Table 4:** MPT Parameter Estimates for both gist and non-gist conditions across the training and control groups in Experiment 2

Parameters	Gist condition		Non-gist condition	
	Training group	Control group	Training group	Control group
Vi	.60 [.35, .80]	.51 [.26, .71]	.75 [.63, .85]	.68 [.56, .78]
Vr	.10 [.00, .28]	.04 [.00, .14]	.07 [.00, .25]	.12 [.00, .37]
Gi = Gr	.86 [.80, .90]	.88 [.83, .92]	.77 [.68, .85]	.81 [.70, .91]
a	.32 [.19, .48]	.34 [.21, .50]	.38 [.24, .56]	.26 [.15, .43]
ab	.11 [.00, .44]	.06 [.00, .16]	.11 [.00, .51]	.10 [.01, .26]
b	.05 [.02, .09]	.08 [.04, .12]	.04 [.01, .08]	.08 [.03, .13]

Vi = verbatim trace is retrieved given an intact probe; Vr = verbatim trace is retrieved given a related probe; Gi = gist trace is retrieved given an intact probe; Gr = gist trace is retrieved given a related probe; a = guessing “intact” on retrieving the gist; ab = guessing “intact” on entering state b; b = guessing “intact” or “related” when gist is not retrieved

*Structural equation modeling.* Four eye movement parameters (i.e., FD, FD_P, FIX, and FIX_P) constructed a latent variable “interference” reflecting older adult’s fixations on the interfering word AOI, with standardized estimates of 0.96, 0.98, 0.99, and 0.99 respectively. Acute-training was negatively correlated with interference (*β* = − 0.32, *p* = 0.01) and had no direct association with “Intact” response in related probes (*β* = 0.18, *p* = 0.12) and memory ACC (*β* = − 0.06, *p* = 0.49). Interference was positively correlated with “Intact” response in related probes (*β* = 0.50, *p* < 0.001), but negatively correlated with ACC (*β* = − 0.50, *p* < 0.001).

**Fig. 7:**
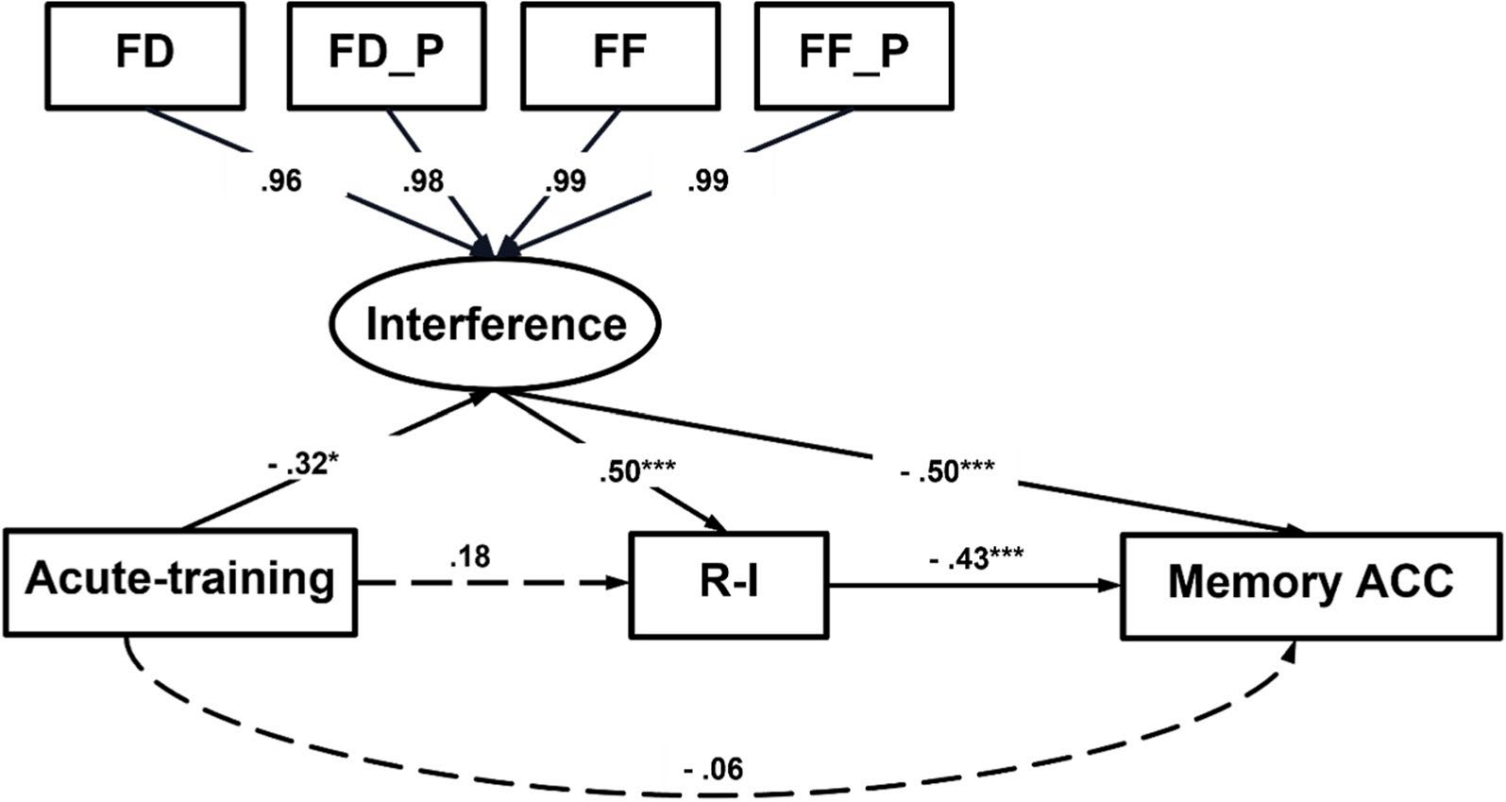
Structural equation modelling. Acute-training = the acute-training (2) and control group (1); Interference is eye movement parameters in the interfering word AOI. FD = fixation duration; FD_P = the proportion of fixation duration to total duration; FF = fixation frequency; FF_P = the proportion of fixation frequency to total frequency; R-I = the proportion of “intact” responses to related probes; Memory ACC = the accuracy of associative memory task. *p < 0.05. ***p < 0.001

Figure 7 shows the path analyses, which revealed a chain mediation model in which acute-training affected memory ACC through a chain mediation pathway, with interference reflected by eye parameters and “Intact” response in related probes acting as mediators (*β* = 0.07, *p* = 0.04). The model had a good fit (i.e., χ^2^ /df = 1.25, CFI = 0.997, TLI = 0.994, SRMR = 0.011 and RMSEA = 0.065). In addition, a mediation model was also established in which acute-training affected memory ACC through affecting interference (*β* = 0.16, *p* = 0.02). Detailed model results are provided in Table S4 in the Supplemental Material.

In summary, structural equation modeling incorporating the results of eye movements was used to explore the potential mechanism underlying memory difference between the two groups. The results indicated acute-training effects on memory ACC by first decreasing attention to interference, which then led to a reduction in “Intact” response in related probes error response. This chain mediation results again highlight the key role of “Intact” response in related probes errors in older adult’s associative memory differences.

### Discussion

In Experiment 2, we explored whether and how acute-training inhibitory function could ameliorate the effect of interfering information on associative memory in older adults. We found that while there were no significant differences in memory performance between the acute training and control groups, there was an encouraging possibility indicating that acute training inhibitory function could improve the impaired associative memory of older adults when distraction is present at encoding. This improvement was observed through a pathway revealed by eye-tracking technology. Specifically, acute training of older adults’ inhibitory function diminished the processing of interfering information, which allowed older adults to make less inaccurate responses (i.e., make wrong choice to “Intact” under related probes, “Intact” response in related probes) and eventually enhanced their accuracy in the associative memory task.

## General discussion

In the current study, we systematically studied the role of inhibitory function in associative memory decline in older adults, and whether and how it is possible to acutely train inhibitory function to improve associative memory in older adults. We also considered the effect of interfering stimuli properties, and used modified Stroop task to directly measure inhibitory function. We did not limit ourselves to measuring a single memory performance (i.e., accuracy) as in previous studies, but focused on verbatim and gist memory based on fuzzy trace theory (Brainerd & Reyna, 1990, 2004; Reyna & Brainerd, 1995). The eye-tracking technology was deliberately chosen to precisely capture the benefits of acute inhibitory training and to investigate the behavioral mechanisms underlying associative memory improvement in older adults. Using various analytical methods including the MPT model, conditional process analysis, and structural equation modelling (Fig. 7), this study arrived at the four main findings about the inhibitory deficit in associative memory and its plasticity in older adults. Experiment 1 indicates that (1) gist representations more than non-gist interfered with associative memory in older adults, and (2) inhibitory function deficits mediated the relationship between age and associative memory decline. Experiment 2 revealed that (3) acute inhibitory training did not significantly improve associative memory accuracy in the training group compared to the control group, but (4) older adults in the acute training group showed shorter fixation durations and frequencies in the interference region of interest, leading to better associative memory.

## Inhibitory deficits exacerbate associative memory deficits in older adults

Inhibitory function deficit mediated the relationship between age and associative memory decline, which is consistent with Hypothesis 2. Our results reveal an inhibition-related mechanism for the decline in associative memory in older adults. Specifically, older adults, due to impaired inhibitory function, form more gist-related associations of interfering information with the target, and respond inaccurately, thus leading to the decline of associative memory, which is reflected by the chain mediation between age group and associative memory accuracy in Experiment 1. As individuals age, inhibitory deficits exacerbate associative memory decline in older adults, leaving them increasingly vulnerable to interference (Hasher, 2015; Hasher & Zacks, 1988). Consequently, they tend to create more false associations with interfering information during the memory encoding stage. This allows an increase in inaccurate responses during the retrieval of mnemonic associations, ultimately resulting in reduced associative memory accuracy.

Compared to the younger group, the older adult group had a lower rate of accuracy on the associative memory task. These also provide evidence for the associative deficit hypothesis that older adults’ associative memory decline is due to their impaired ability to make connections between episodic components (Naveh-Benjamin, 2000; Naveh-Benjamin et al., 2003, 2004; Old & Naveh-Benjamin, 2008). Older adults suffered more interference from gist-related information and made more inaccurate “intact” response to related probes based on their gist memory, which was revealed by an interaction effect of age groups and interference condition on “Intact” response in related probes in Experiment 1. This indicates that older adults’ overreliance on gist memory exacerbated the adverse effects of interference information, particularly gist-related interference, due to their inhibitory deficits and the less specificity of gist memory (Abadie et al., 2021; Greene & Naveh-Benjamin, 2020). This explains why older adults made more inaccurate responses in related probes and subsequently exhibited associative memory deficits.

In addition, the “Intact” response in related probes response error results reveal that older adults are more likely to respond "intact" to related probes in the gist interference condition than in the non-gist interference condition. These results align with the findings regarding the guessing parameter a in the MPT model. This parameter indicates that older adults tend to guess "intact" more frequently than younger adults when retrieving gist memory in the gist interference condition. This error response or guessing strategy in older adults may reflect that the related words came to mind more often during encoding in the gist interference condition. This leads to a false feeling of familiarity that older adults may have difficulty inhibiting and may rely on more during retrieval (Rhodes et al., 2008).

## Gist-related interferences form more but inaccurate association in older adults due to hyper-binding

Gist representation more than non-gist interfered with associative memory in older adults, which is consistent with Hypothesis 1b. The MPT model showed that older adults showed more gist memory in the gist interfering word condition (i.e., gist representation) than in the non-gist interfering word condition, whereas both verbatim memory and gist memory were unaffected by these two conditions in younger adults. Immunity to interfering information in the younger cohort reflects the well-developed inhibitory function of younger people, which could effectively suppress the destructive effect of interfering information invasion on memory. The inhibitory function index measured by the Stroop task also supported this conclusion, as the inhibitory function index of younger people was significantly higher than that of older people.

There was no difference in older adults’ ability to access the verbatim representations of target items in the gist and non-gist interference conditions. However, upon failing to access these representations, older adults were more likely to be able to remember the gist of the target items in the gist versus non-gist interference condition. Indeed, gist interference condition facilitated better gist memory. The strengthened gist memory observed in older adults might be a byproduct of impaired inhibitory control. Even although gist memory increased, it primarily reflects a failure to inhibit interference from gist-related words that should have been ignored. These gist memory benefits can be attributed to hyper-binding in older adults. According to the hyper-binding view, older adults encode seemingly unrelated co-occurrences in the environment due to inhibitory deficits and transfer this knowledge to subsequent tasks (Campbell & Davis, 2024; Campbell et al., 2010). The hyper-binding process in older adults is considered to be a relatively automatic and implicit process (Campbell & Davis, 2024), which explains why older adults showed significantly more gist memory in the gist interference condition, even though they were asked to ignore the interference words following the task instruction. Furthermore, our results revealed that when interfering information had semantic relationships with targets, hyper-binding was exacerbated in older adults. This suggests that gist representation may require fewer cognitive resources for processing and integration into gist memory (Reyna & Mills, 2007; Greene & Naveh-Benjamin, 2023a, 2023b). Notably, an increase in gist memory caused by hyper-binding is not always beneficial. According to the MPT model, extraction of gist representations has the probability to lead to incorrect responses (“1-*a”* for intact probe and “*a”* for related probe), whereas extraction of verbatim representations typically leads to correct responses to probes. Therefore, compared to verbatim memory, gist memory is more robust with age, but less accurate. Older adults’ over-reliance on gist memory is a possible reason why they make more inappropriate responses than younger adults. This is also supported by our finding that older adults still had significantly lower accuracy than younger adults, even though they formed more gist memory because of hyper-binding.

Older adults demonstrated greater gist memory in the gist interfering condition than in the non-gist interfering condition. However, verbatim memory was not affected by the interference conditions. Note that we instructed participants to ignore the interfering word as much as possible, which would inevitably weaken the original influence of the interfering word. However, even after the interference effect was attenuated by the inhibitory function, the dissociation results showed that older adults’ gist memory was affected, and their verbatim memory was not. The divergent patterns observed in the effects of interfering information on verbatim and gist memory in older adults suggest three points. First, impairments in inhibitory function in older adults result in their inability to suppress the influence of interfering information, a phenomenon supported by previous research (Hasher, 2015; Hasher & Zacks, 1988) and the inhibitory function index in the current study. Second, gist more than non-gist representations interfered with associative memory in older adults, which eventually enhanced their gist memory. This is likely to be attributed largely to the fact that gist representations are more automatic and easier to form, which require fewer attentional resources to integrate into gist memory (Brainerd & Reyna, 1990, 2002; Greene & Naveh-Benjamin, 2023a). In addition, older adults’ over-reliance on gist memory (Greene & Naveh-Benjamin, 2023a, 2023b) and hyper-binding (Campbell & Davis, 2024; Campbell et al., 2010) aggravated the gist interfering effect. Third, gist memory cannot be considered merely a by-product of verbatim memory. Although both types of memory are episodic, they capture different details of the episode according to fuzzy trace theory (Brainerd & Reyna, 2004), as shown by the divergent patterns in our results. This finding adds substantive evidence to the ongoing debate on the dichotomy between verbatim and gist memory. In this debate, fuzzy trace theory argues that verbatim and gist memory are distinct mnemonic entities (Brainerd & Reyna, 2004; Wolfe, 2021), whereas the gist macroprocessor framework posits that gist memory emerges because of verbatim memory processes (Kintsch, 1988; Kintsch & van Dijk, 1978; Perfetti, 2007).

## Implications of inhibitory function training for associative memory improvement

The acute inhibitory training did not significantly enhance the associative memory of older adults in the training group, which did not align with hypothesis 3. We suspected that this was mainly due to the tiny effect of single, acute inhibitory training. Although we ensured that each older adult should have a high accuracy on the training task, with an average accuracy of over 90%, in order to increase the training effects, acute short-term inhibitory training was still not efficient and sufficient to induce memory improvement. This result suggests that the memory-related effect of inhibitory training depends on the time and degree of training. Long-term and repeated training, rather than acute and single training, is crucial for the training effects (Diamond & Ling, 2016; Lin et al., 2024; Nguyen et al., 2019; Zhao et al., 2018).

Additional exploration analyses based on our eye-tracking data were also conducted to investigate the feasibility of an inhibitory training effect on associative memory, targeting Hypothesis 4. We found a desirable neuropsychological pathway by structural equation modelling that acute-training on inhibitory function reduced interference processing in the interfering areas in older adults, and then reduced their inaccurate response related to interference, thereby improving associative memory. Using eye-tracking technology, we explored the behavioral mechanisms underlying the effectiveness of inhibitory training by optimizing the distribution of attentional resources. According to the resource limitation theory (Kahneman, 1973), the cognitive system has limited resources, and when performing a task, individuals must efficiently allocate these limited cognitive resources, including attention, working memory capacity, and other cognitive abilities (Nieznański & Obidziński, 2019). One of the core concepts of this theory is competitive resource allocation, in which multiple tasks compete for limited cognitive resources. For older adults, there is a reduction in or an inequitable allocation of resources owing to cognitive aging (Craik, 2002; Craik & Bialystok, 2006; Craik & Rose, 2012), indicating that itself in older adults being more susceptible to performance decline in complex memory tasks than younger adults. The inhibitory function involves the inhibition of interfering information, which is particularly important in memory tasks because older adults are more susceptible to external distractions (Hasher, 2015; Hasher & Zacks, 1988; Yang et al., 2022). Inhibitory training modulates older adults’ cognitive resources, allowing them to allocate their resources more efficiently and reduce their sensitivity to interfering information. This is supported by the current study’s evidence that older adults in the acute-training group had less attentional resource allocation to interfering areas, and thus performed better on associative memory. Expressly, the feasibility of inhibitory training to improve associative memory lies in its ability to improve cognitive control and optimize older adults’ use of cognitive resources, which is extremely important in the context of limited cognitive resources that decline with age.

## Limitation and future direction

First, we acknowledge that the lower education levels among older adults could introduce confounding factors when examining age-related differences. However, we addressed this by controlling for years of education in all our analyses, including the ANCOVA for response proportion, MPT modeling, and mediation model analyses. In addition to these analytical approaches, we deliberately adjusted the task difficulty to favor older adults in the study, aiming to mitigate these educational disparities that are not pertinent to our research objectives. Second, our associative memory paradigm differed from previous studies in that we did not use recombined test probes, which could have potentially undermined the efficacy of the associative memory. This design was driven by the lower education and memory of older adults in our experiments, prompting us to adjust the experimental difficulty. Nevertheless, the present pattern of memory results was similar to previous studies evidenced by a pattern of intact > related probes during associative recognition in older adults, indicating the formation of associative memory in older adults. Third, the benefits of acute inhibitory training were not reflected in memory performance, suggesting that the single-session acute training effect is subtle and insufficient for a significant associative memory improvement. Future direction is to explore the effects of long-term inhibitory intervention on associative memory decline in older adults. Fourth, in the gist condition, the test words of the related probe were presented as gist interference words that supposed be ignored in the study phase. In contrast, the words in the non-gist condition were not presented, but were semantically related to the target words. To address this issue, we estimated a new MPT model with a phantom recollection parameter that represents recollection of the interference words (Greene & Naveh-Benjamin, 2024; Stahl & Klauer, 2009). The results showed no statistically significant difference in the phantom recollection parameters between the two interference conditions. This suggests that the confounding effect of repeatedly presenting of words could be limited. Fifth, MPT models, based on fuzzy trace theory, provide estimates of the probability that participants access their verbatim or gist representations, as well as response bias estimates. However, caution is warranted when interpreting these model estimates. This is because the MPT model is a measurement tool by nature. Although it can quantitatively model dissociable memory processes based on theoretical assumptions, it does not provide direct evidence of underlying psychological processes nor explain how these processes emerge. Although our MPT models fit the current data well, more studies are needed to assess and validate the psychological implications of these MPT parameters, such as different guessing strategies or phantom recollections.

## Conclusion

In conclusion, inhibitory function plays a mediating role in age-related associative memory decline, as well as its plasticity in this association. Due to impaired inhibitory function, gist representation more than non-gist interferes with associative memory in older adults, which made them form more inaccurate associations during memory encoding, or hyper-binding, ultimately leading to increased Interference at associative memory retrieval. In addition, it has the possibility to train inhibitory functions to improve associative memory in older adults, and the behavioral mechanism behind this is meant to optimize the allocation of attentional resources in older adults. Our study provides direction for clinical interventions targeting associative memory improvement.

### Public significance statement

Memory depends on both retaining relevant information and filtering out what is irrelevant. Associative memory—the ability to link elements of an episode—declines with age, partly due to reduced ability to inhibit familiar but irrelevant information. However, how this inhibitory deficit unfolds in real time, and whether short-term interventions can improve it, remains unclear. This is important for older adults, who often face interference from similar or overlapping memories, such as medication routines or recognizing various traffic signs. In two experiments, we investigated whether inhibitory function mediates age-related deficits in associative memory and whether acute inhibitory training can alleviate such interference. Experiment 1 revealed that older adults were more affected by gist-related interference, indicating a tendency to “hyper-bind” to similar yet irrelevant information. Experiment 2 used eye-tracking to examine the effects of acute inhibitory training. This training helped older adults ignore interference more effectively, as evidenced by reduced gaze duration and frequency on interfering regions, which are markers of more efficient attentional control. These improvements in attention were predictive of enhanced associative memory. Our findings suggest that inhibitory function contributes to age-related memory deficits and exhibits short-term plasticity. Training older adults to shift their attention away from irrelevant information could be a promising strategy for supporting their cognitive health. In practice, these findings suggest that interventions such as simplifying medical labels or safety warnings—reducing environmental “noise”—may be more effective than merely urging older adults to “remember better.”

## Supplementary Information

Additional file1 (DOC 430 KB)

## Data Availability

To support transparency and openness, all data, materials, and analysis scripts are available at OSF: https://osf.io/793ge/. Experiment 1 was pre-registered with Aspredicted: https://aspredicted.org/tq9uq.pdf. Experiment 2 has been pre-registered at https://aspredicted.org/5hc9a.pdf.
