# Supplementary material for: The role of inhibitory function in associative memory among older adults and its plasticity
Source: Cogn Res Princ Implic. 2025 Nov 16;10:78. doi: 10.1186/s41235-025-00688-5 (PMC12620347; doi:10.1186/s41235-025-00688-5)
Supplement: Supplementary file 1 — Additional file1 (DOC 430 KB) [file 41235_2025_688_MOESM1_ESM.doc]

**Supplemental Material**

**MPT model.**

The MPT model can measure the verbatim memory and gist memory in the recognition task, which reflects in the corresponding parameter V and G. We used Greene's MPT model (Greene & Naveh-Benjamin, 2020) with one modified parameters: ab instead of a in the previous Stahl & Klauer’s MPT model (Stahl & Klauer, 2008). Greene’s model assumed that participants’ bias to respond “intact” can change depending on whether or not the gist is retrieved, while the original SCR paradigm (Stahl & Klauer, 2008) included a a parameter for responding “intact”, regardless of gist. We used the TreeBUGS package for R (Heck et al., 2018; Team, 2020) to separately run the model in both age groups. We set the default priors and MCMC chains (n.chains = 3) from the TreeBUGS package. 60,000 iterations (n.iter=60000) were run with a 15000 adaptation (n.adapt=15000) period and 10000 burn-in period (n.burnin=10000). The p-values of T1 and T2 statistics were used to represent whether the model satisfactorily fit the data. The model is considered adequate about the deviations quantified if p is not small (Klauer, 2010). All the MCMC chains converged on a stable posterior distribution for each parameter (R-hat values < 1.05).

**Goodness of model fit**

These posterior predictive checks can be performed graphically by plotting the observed mean frequencies against the distribution of mean frequencies that are sampled from the hierarchical model, using the posterior samples as data-generating parameters. Figure s1 shows the resulting plots of both Stahl's and Greene's MPT models, which indicate that Greene's model fits our data better, because the observed and predicted values differ less than Stahl's.


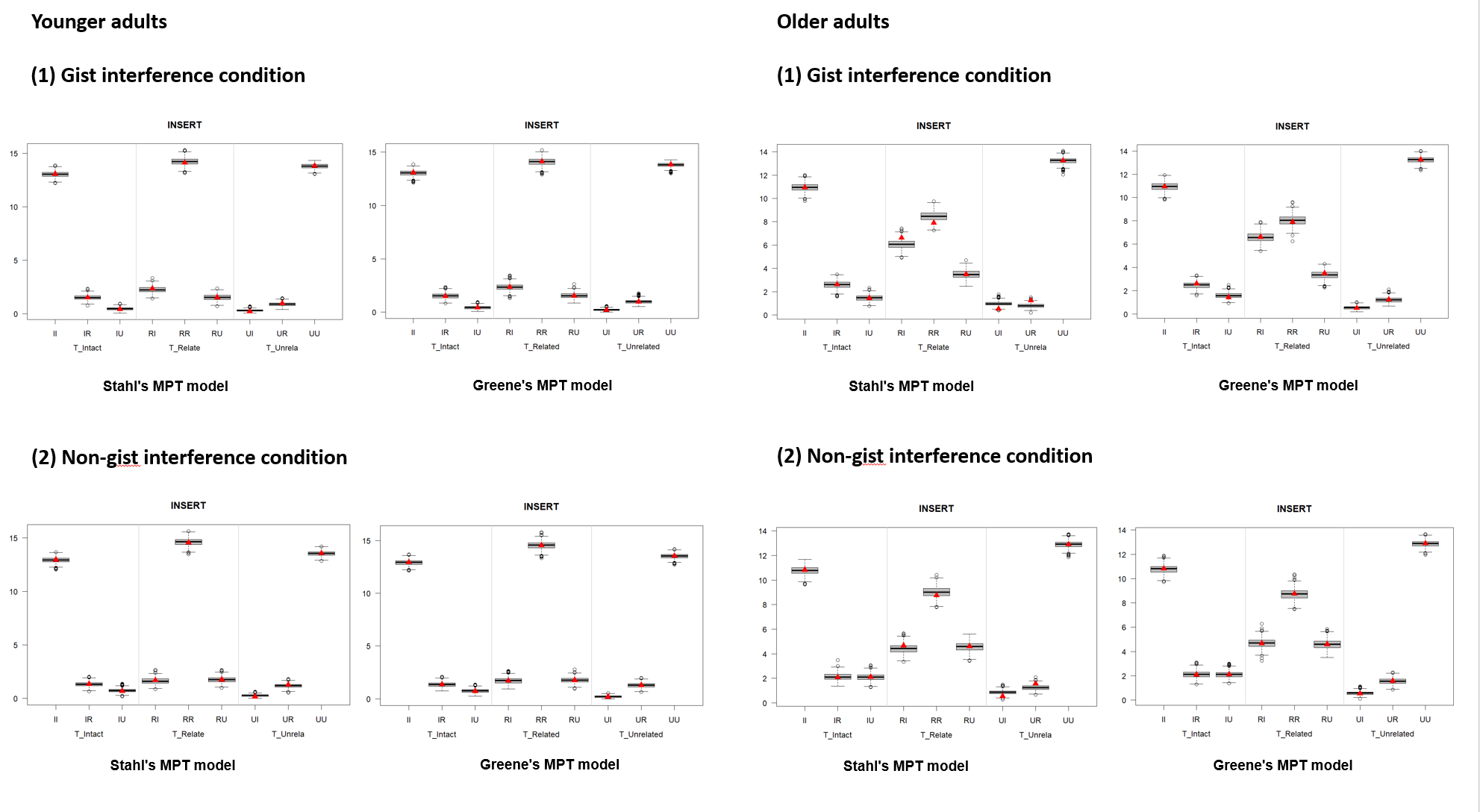


**Figure S1.** Posterior predictive checks plots for younger and older adults under gist and non-gist interference conditions about Greene’s MPT and original MPT models.

Posterior predictive p values (PPP) which include T1 and T2 p values are used as a quantitative assessment of model fit. Whereas small PPP values close to zero indicate insufficient model fit, larger values indicate satisfactory model fit. All p-values of T1 and T2 for Stahl's MPT models are smaller than those for Greene's model, which also means Greene’s model fits our data better. The T1 and T2 statistical p-values for Stahl's MPT models are 0.36 and 0.39 for younger adults in the gist interference condition, whereas these are 0.49 and 0.43 for Greene's models; 0.38 and 0.38 for younger adults in the non-gist interference condition, whereas these are 0.49 and 0.38 for Greene's model; 0.01 and 0.06 for older adults in the gist interference condition, whereas these are 0.45 and 0.50 for Greene's models; 0.07 and 0.42 for older adults in the non-gist interference condition, whereas these are 0.49 and 0.44 for Greene's model.

**The Posterior probability distribution of *G*i**

Figure S2 blow shows the posterior probability distribution of Gi for older and younger adults in gist and non-gist interference conditions, which directly reflects the different distribution patterns of gist and non-gist interference conditions between young and older adults. Although Figure 4 shows remarkably similar directional patterns of gist interference effects on parameter G between age groups, this result indicated the the true difference of G between gist and non-gist interference condition in older adults.


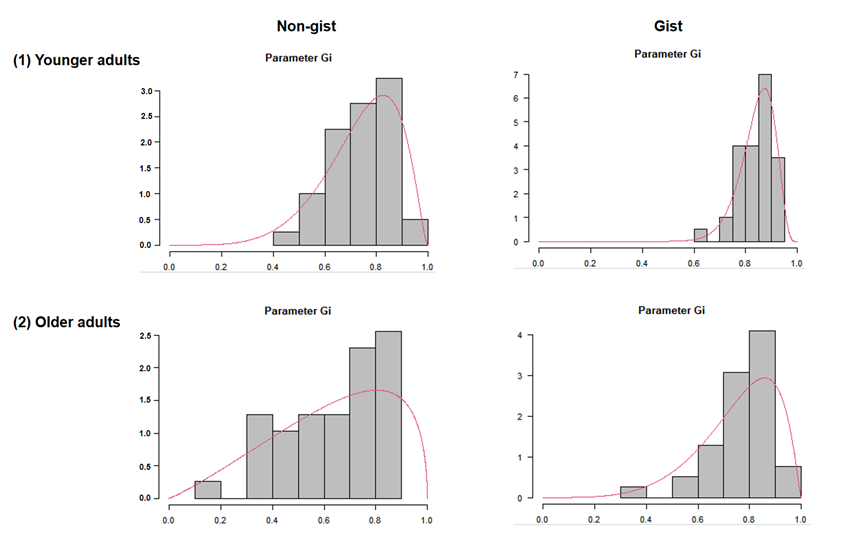


**Figure S2.** Posterior probability distribution of Gi for older and younger adults in gist and non-gist interference conditions.

**Mediation model analyses of Experiment 1**

**Table S1.** Results of chain mediation model in Experiment 1.

|  | β | SE | *t* | *p* | LLCI | ULCI |
| --- | --- | --- | --- | --- | --- | --- |
| Model 1 | | | | | | |
| Outcome: IF | | | | | | |
| Predictors: | | | | | | |
| Age | -0.38 | 0.11 | -3.63 | <0.001 | -0.59 | -0.17 |
| Model 2 | | | | | | |
| Outcome: RI | | | | | | |
| Predictors: | | | | | | |
| Age | 0.51 | 0.10 | 5.19 | <0.001 | 0.31 | 0.70 |
| IF | -0.21 | 0.10 | -2.12 | 0.04 | -0.40 | -0.01 |
| Model 3 | | | | | | |
| Outcome: ACC | | | | | | |
| Predictors: | | | | | | |
| Age | -0.33 | 0.10 | -3.29 | 0.00 | -0.53 | -0.13 |
| IF | -0.10 | 0.09 | -1.15 | 0.25 | -0.28 | 0.07 |
| RI | -0.53 | 0.10 | -5.23 | <0.001 | -0.73 | -0.33 |
| Conditional indirect effect | |  | *β* | *Boot SE* | *Boot LLCI* | *Boot ULCL* |
| Age → IF → RI → ACC | |  | -0.04 | 0.03 | -0.12 | -0.01 |
| Age → IF → ACC | |  | 0.04 | 0.04 | -0.04 | 0.14 |
| Age → RI → ACC | |  | -0.27 | 0.07 | -0.43 | -0.15 |
| Conditional direct effect | | *β* | *SE* | *t* | *LLCI* | *ULCL* |
| Age on ACC |  | -0.33 | 0.10 | -3.29 | -0.53 | -0.13 |

Note: Bootstrap sample size = 5000. Age = older and younger adult groups; IF = inhibitory function index from Stroop task; RI = the proportion of “intact” response in related probes; ACC = the accuracy of associative memory; LL = lower limit; CI = confidence interval; UL = upper limit.

**Table S2.** Estimation of indirect effects of mediation models.

| Mediation models | *Effect* | *Boot SE* | *Boot LLCI* | *Boot ULCL* |
| --- | --- | --- | --- | --- |
| Group → IF → IR → ACC | 0.02 | 0.02 | -0.01 | 0.06 |
| Group → IF → IU → ACC | -0.03 | 0.03 | -0.09 | 0.01 |
| Group → IF → RU → ACC | -0.08 | 0.06 | -1.46 | 0.14 |
| **Group → IF → UI → ACC** | **0.04** | **0.02** | **0.01** | **0.07** |
| Group → IF → UR → ACC | -0.01 | 0.02 | -0.04 | 0.02 |
| **Group → IF → IR → Vm** | **-0.03** | **0.02** | **-0.06** | **-0.00** |
| Group → IF → IU → Vm | -0.02 | 0.02 | -0.07 | 0.01 |
| Group → IF → RI → Vm | 0.02 | 0.01 | -0.00 | 0.04 |
| Group → IF → RU → Vm | -0.01 | 0.01 | -0.04 | 0.02 |
| Group → IF → UI → Vm | 0.02 | 0.01 | -0.00 | 0.04 |
| Group → IF → UR → Vm | -0.00 | 0.01 | -0.03 | 0.01 |
| Group → IF → IR → G | -0.01 | 0.01 | -0.03 | 0.00 |
| Group → IF → IU → G | -0.05 | 0.04 | -0.14 | 0.01 |
| Group → IF → RI → G | -0.01 | 0.01 | -0.02 | 0.00 |
| Group → IF → RU → G | -0.01 | 0.03 | -0.09 | 0.05 |
| **Group → IF → UI → G** | **0.02** | **0.01** | **0.00** | **0.05** |
| Group → IF → UR → G | -0.00 | 0.01 | -0.03 | 0.02 |

Note: Education were controlled as a covariate. Bootstrap sample size = 5000. Group = older and younger adult groups; IF = inhibitory function index from Stroop task; IR= the proportion of “related” response in intact probes; IU= the proportion of “unrelated” response in intact probes; RI = the proportion of “intact” response in related probes; RU= the proportion of “unrelated” response in related probes; UI = the proportion of “intact” response in unrelated probes; UR= the proportion of “related” response in unrelated probes;ACC = the accuracy of associative memory; Vm = verbatim memory (average of Vi and Vm) from MPT model; G = gist memory from MPT model; LL = lower limit; CI = confidence interval; UL = upper limit.

**Experiment 2**

**Flanker task**

During the Flanker task, the participants were required to make a judgement about the central arrow, suppress interference from the direction of the arrows on either side, decide whether the central arrow was pointing left or right, and make a corresponding button response. There were two conditions for the task: (1) a consistency condition in which the central arrow was aligned with the orientation of both sides (e.g., ">>>>>" or "<<<<<"); and (2) an inconsistency condition, in which the central arrow was not aligned with the orientation of both sides (e.g., ">><>>" or "<<><<"). The participants received feedback on their current accuracy and reaction time after each trial. Successful intervention in the inhibitory function was determined by an accuracy rate exceeding 90%. In cases where the intervention was unsuccessful, additional rounds of the Flanker task were administered. Upon successful intervention, the participants immediately proceeded to the memory test. The participants in the control group did not perform the Flanker task.

**Group differences**

***Proportion of correct response.*** The results of Experiment 2 showed no significant differences among the groups for the three probes (see Figure S3).


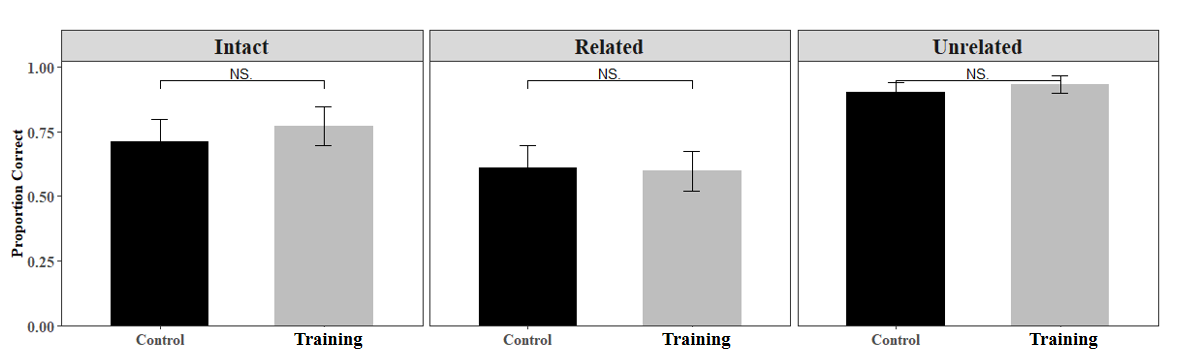


**Figure S3.** Proportion of correct responses in Experiment 2. Note: NS. *p* > 0.05.

**Table S3. Demographic information of training and control groups in Experiment 2**

|  | Training group（n=30）  M±SD | Control group（n=29）  M±SD | t | p |
| --- | --- | --- | --- | --- |
| Age | 67.27±4.02 | 67.90±4.89 | 0.54 | 0.59 |
| Gender（male/female） | 2/28 | 2/27 | 2.29e-31a | 1 |
| Educational years | 10.23±2.62 | 11.21±2.66 | 1.41 | 0.16 |
| MMSE | 28.93±1.17 | 28.93±0.92 | -0.01 | 0.9 |

Note: a represents the value of χ2 test；MMSE = Mini-mental State Examination.

***MPT results.*** The results showed no significant differences in any of the MPT model parameters between the acute-training and control groups. Table 4 shows the results of the parameter estimates for each group under gist and non-gist conditions. Figure S4a presents the group differences for each parameter obtained by subtracting the posterior samples of the control group from those of the training group to obtain a credibility interval. Figures S4b and S4c present the group differences for each parameter under the gist and non-gist conditions, respectively. The results showed no significant differences in any of the MPT model parameters between the intervention and control groups.


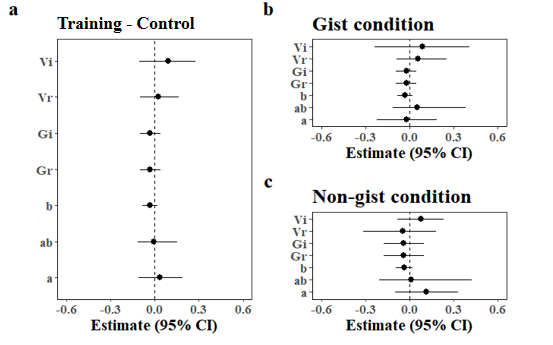


**Figure S4.** The MPT figures for group difference of Experiment 2. Forest plots depict the difference score (Training minus control) for each parameter: (a) in the overall condition; (b) in the gist condition; (c) in the non-gist condition.

**Table S4.** Estimation of indirect effects of structural equation modelling.

| Mediation models | *Estimate* | *S.E.* | *Z*-value | *P*( >|*Z*| ) |
| --- | --- | --- | --- | --- |
| Group → Interference → RI → ACC | 0.07 | 0.04 | 1.99 | 0.04 |
| Group → Interference → ACC | 0.16 | 0.07 | 2.32 | 0.02 |
| Group → RI → ACC | -0.08 | 0.06 | -1.46 | 0.14 |

**Note:** Group = the acute-training versus control groups; Interference = a latent variable from four eye movement indices; RI = the proportion of “intact” response in related probes; ACC = the accuracy of associative memory.

**Reference**
